# The role of extent of resection in gliosarcoma compared to glioblastoma—A volumetric matched-cohort assessment of residual contrast-enhancing tumor

**DOI:** 10.1093/noajnl/vdag235

**Published:** 2026-11-10

**Authors:** Obada T Alhalabi, Philipp Sievers, Manuel Kaes, Ahmad Gamal Abo Arab, Kirill Mironov, Henri Oldashi, Ahmed Nor Gareib, Johanna Krämer, Christel Herold-Mende, Marianne Schell, Tobias Kessler, Felix Sahm, Bogdana Suchorska, Sandro M Krieg, Sebastian Ille

**Affiliations:** Department of Neurosurgery, Heidelberg University Hospital, Ruprecht-Karl-University, Heidelberg, Germany; Clinical Cooperation Unit Neurooncology, German Cancer Research Center (DKFZ), Heidelberg, Germany; Department of Neuropathology, Institute of Pathology, Heidelberg University Hospital, Heidelberg, Germany; Clinical Cooperation Unit Neuropathology, German Consortium for Translational Cancer Research (DKTK), German Cancer Research Center (DKFZ), Heidelberg, Germany; Department of Neurosurgery, Heidelberg University Hospital, Ruprecht-Karl-University, Heidelberg, Germany; Department of Neurosurgery, Heidelberg University Hospital, Ruprecht-Karl-University, Heidelberg, Germany; Department of Neurosurgery, Heidelberg University Hospital, Ruprecht-Karl-University, Heidelberg, Germany; Clinical Cooperation Unit Neurooncology, German Cancer Research Center (DKFZ), Heidelberg, Germany; Department of Neurosurgery, Heidelberg University Hospital, Ruprecht-Karl-University, Heidelberg, Germany; Clinical Cooperation Unit Neurooncology, German Cancer Research Center (DKFZ), Heidelberg, Germany; Department of Neurosurgery, Heidelberg University Hospital, Ruprecht-Karl-University, Heidelberg, Germany; Department of Neurosurgery, Heidelberg University Hospital, Ruprecht-Karl-University, Heidelberg, Germany; Division of Experimental Neurosurgery, Department of Neurosurgery, University Hospital of Heidelberg, Heidelberg, Germany; Department of Neuroradiology, University Hospital Heidelberg, Heidelberg, Germany; Clinical Cooperation Unit Neuropathology, German Consortium for Translational Cancer Research (DKTK), German Cancer Research Center (DKFZ), Heidelberg, Germany; Department of Neurology and Neurooncology Program, National Center for Tumor Diseases, University Hospital Heidelberg, Heidelberg, Germany; Department of Neuropathology, Institute of Pathology, Heidelberg University Hospital, Heidelberg, Germany; Clinical Cooperation Unit Neuropathology, German Consortium for Translational Cancer Research (DKTK), German Cancer Research Center (DKFZ), Heidelberg, Germany; Department of Neurosurgery, Heidelberg University Hospital, Ruprecht-Karl-University, Heidelberg, Germany; Department of Neurosurgery, Heidelberg University Hospital, Ruprecht-Karl-University, Heidelberg, Germany; Department of Neurosurgery, Heidelberg University Hospital, Ruprecht-Karl-University, Heidelberg, Germany

**Keywords:** extent of resection, glioblastoma, gliosarcoma, gross total resection, volumetric analysis

## Abstract

**Background:**

The extent of resection (EOR) of contrast-enhancing tumor is an established determinant of survival in glioblastoma (GB). However, its prognostic role in gliosarcoma (GS), a histopathologically distinct GB subtype, remains poorly characterized. This study investigated the influence of EOR on survival in GS compared to GB.

**Methods:**

In this retrospective study, we analyzed clinical, surgical, and volumetric MRI data—including early postoperative MRI (within 48 h)—from patients undergoing resection for histopathologically confirmed primary GS between 2007 and 2023. A cohort of 130 GB patients served as a reference. Patients developing GS upon recurrence of primary GB were excluded.

**Results:**

Of 63 patients screened, 49 met the inclusion criteria. Gliosarcoma patients presented with significantly larger mean preoperative contrast-enhancing tumor volumes than GB patients (32.1 vs 22.4 mL, *P* = .021), yet achieved comparable rates of gross total resection (mean residual tumor volume: 0.09 vs 0.12 mL, *P* = .435). Median overall survival was 720 days in GS and 648 days in GB patients. Complete resection (0 mL residual tumor) conferred a more pronounced survival benefit in GS than GB: median overall survival was 720 vs 402 days for GS patients with 0 mL vs >0-1 mL residual tumor (*P* = .022), compared to 713 vs 510 days in GB (*P* = .042). A matched cohort analysis (*n* = 54) confirmed this differential benefit.

**Conclusions:**

Complete resection may confer a greater survival benefit in GS than in GB, suggesting that EOR influences prognosis differently in this histopathologically distinct subtype and supporting a tailored surgical approach to GS.

Key Points

- Gliosarcoma patients present with larger tumor volumes than glioblastoma, yet are amenable to comparable rates of gross total resection.
- Extent of resection may influence survival differently in gliosarcoma, supporting a distinct surgical approach to this tumor subtype.

Importance of the Study

The influence of extent of resection (EOR) on survival in gliosarcoma (GS) has remained poorly defined, as this histopathologically distinct subtype of glioblastoma is rarely studied in isolation. This study demonstrates that despite presenting with significantly larger contrast-enhancing tumor volumes, GS patients achieve gross total resection at rates comparable to glioblastoma, and that complete resection—defined as 0 mL residual tumor volume—confers a markedly greater survival benefit in GS than in glioblastoma. These findings were consistent in both the full cohort and a matched analysis controlling for demographics and O-6-methylguanine-DNA methyltransferase status. Collectively, our data suggest that EOR may have a distinct prognostic role in GS compared to glioblastoma, providing rationale for investigating whether residual tumor thresholds should be considered differently in GS.

Glioblastoma (GB) is the most common primary malignant brain tumor in adults and remains incurable despite aggressive multimodal therapy.^1^ Standard treatment, including maximal safe resection followed by radiotherapy and temozolomide, provides only a limited survival benefit, with a 5-year survival rate below 10% and a median overall survival (OS) of about 14-16 months in contemporary series.^2-4^ As a result, surgical maximal resection plays a central role in onco-functional-driven disease management.^5^^,^^6^

A universal finding across studies is that the extent of resection (EOR) influences patient survival in GB.^7^^,^^8^ Gross total resection (GTR) of a contrast-enhancing (CE) tumor, resulting in minimal residual tumor volume (RTV), is associated with prolonged OS and progression-free survival (PFS) compared with subtotal or partial resection.^9-11^

Gliosarcoma (GS) is a histological subgroup of IDH-wildtype GB characterized by a combined glial and sarcomatous architecture.^12^ Although observations similar to GB have been made with regard to the effect of EOR on OS in GS,^13^ several studies indicate potential biological and clinical differences in GS to GB, including distinct molecular features and, in some cohorts, worse survival.^14^^,^^15^ Similar to GB, the prognostic relevance of EOR appears to hold in GS patients.^16^^,^^17^ However, it remains unknown whether RTV carries the same prognostic weight in GS as it does in GB or whether the sarcomatous component influences how residual disease impacts survival.

Given these uncertainties, this study investigates whether the prognostic significance of low residual CE tumor burden differs between GB and GS using standardized postoperative volumetric imaging, particularly within the range of near-total resection.

## Methods

### Inclusion Criteria

We included adult patients (age <18 years) with newly diagnosed supratentorial CE and histopathologically diagnosed GS from our institution who underwent resection surgery between 2007 and 2023. The histopathological diagnosis was initially based on routine neuropathological reports generated in clinical practice and was therefore per se blinded to the aims of the present study. Diagnoses were established according to the 2021 WHO Classification of Tumors of the Central Nervous System,^18^ ensuring that all included tumors fulfilled the criteria for GS as a subtype of GB, IDH-wildtype, CNS WHO grade 4. For confirmation, all cases were subsequently reviewed by a second experienced neuropathologist. Patients with GS at recurrence after primary GB were excluded. Overall survival was calculated from the time of surgery until death or the last available follow-up (censorship). Clinical outcomes after surgery were assessed using the Karnofsky performance scale (KPS). Both scores were evaluated in each patient within 7 days before surgery, at discharge, and on post-operative day 7 (POD 7). We also included consecutive adult patients (age > 18 years) with newly diagnosed supratentorial CE and histopathologically confirmed GB from our institution who underwent surgery between 2015 and 2023 as a reference cohort.

#### Tumor Volumetry

Volumetric analysis of preoperative, intraoperative, and early postoperative MRI (epMRI) scans was performed using the Brainlab Elements software SmartBrush (Brainlab, Germany) to measure the absolute tumor volume of the CE tumor regions. The Response Assessment in Neuro-Oncology (RANO) Resect group classification criteria were utilized to determine preoperative preopMRI and RTV on epMRI scans (within 48 h).^4^ T1-CE, in addition to Fluid-attenuated Inverse Recovery (FLAIR)-hyperintense tumor volumes, were measured.

#### Case Matching

To minimize confounding factors between patients with GS and GB, case–control matching was performed. Patients with GS were matched 1:1 to patients with GB who were comparable in their baseline characteristics based on the following covariates: age category (≤60 vs >60 years), O-6-methylguanine-DNA methyltransferase (MGMT) promoter methylation status, and postoperative CE tumor volume (0 mL or >0 and <1 mL). Matching was performed using nearest-neighbor matching without replacement, applying a caliper width of 0.1.

### Statistical Analysis

Differences in continuous variables were analyzed with the Mann–Whitney *U*-test, and differences in proportions were analyzed with the chi-square test or Fisher’s exact test. Variables with possible prognostic effect were assessed using the log-rank test, and all survival curves were visualized as results of the Kaplan–Meier analysis. In general, a 2-sided *P*-value less than .05 was considered statistically significant. Analyses were performed using SPSS Inc. (Chicago, IL, United States) and GraphPad Prism 10.

### Ethics Approval

The ethical committee at the University of Heidelberg approved the protocol for this retrospective analysis under S-374/2024, which has been performed in accordance with the ethical standards laid down in the 1964 Declaration of Helsinki and its later amendments, and for which patient consent was not required.

## Results

### The Nature of GS Patients in a Comprehensive Neurooncology Center

We identified 63 patients with histopathologically confirmed GS treated at our institution between 2007 and 2023. Of these, 49 patients with primary GS were included in the final GS cohort (Figure 1), 14 patients with GS upon recurrence and GB at primary tumor diagnosis were excluded. The mean age at first diagnosis was 61.5 years (SD 12.2; Table 1), and nearly half of the cohort were female (*n* = 23, 46%). The mean preoperative KPS at presentation was 83.3 (SD 10.2) and 81.8 (SD 10.93) on POD 7. O-6-methylguanine-DNA methyltransferase promoter status was available for 43 patients, of whom 17 (34%) were methylated. Gliosarcoma tumors were mainly located in the temporal lobe (*n* = 22, 44%) and the frontal lobe. Less frequent localizations included the parietal lobes (*n* = 6, 12%) and the occipital lobes (*n* = 1, 2%; Table 1). Multifocal involvement was found in 10 cases (20%). Surgical guidance modalities were used heterogeneously. While Intraoperative MRI (iMRI) was employed in 22 patients (44%), 5-aminolevulinic acid (5-ALA) fluorescence guidance was used in 10 (20%), bright light in 12 patients (24%), and both modalities in 1 patient. Following surgery, concomitant temozolomide was administered to 28 patients (56%); 21 patients (42%) received adjuvant temozolomide after completion of initial chemoradiation, whereas adjuvant temozolomide monotherapy was implemented in a smaller number (*n* = 1, 2%). Postoperative irradiation was performed in 41 patients (82%), reflecting the routine application of combined modality therapy in the management of GS.

**Figure 1.**
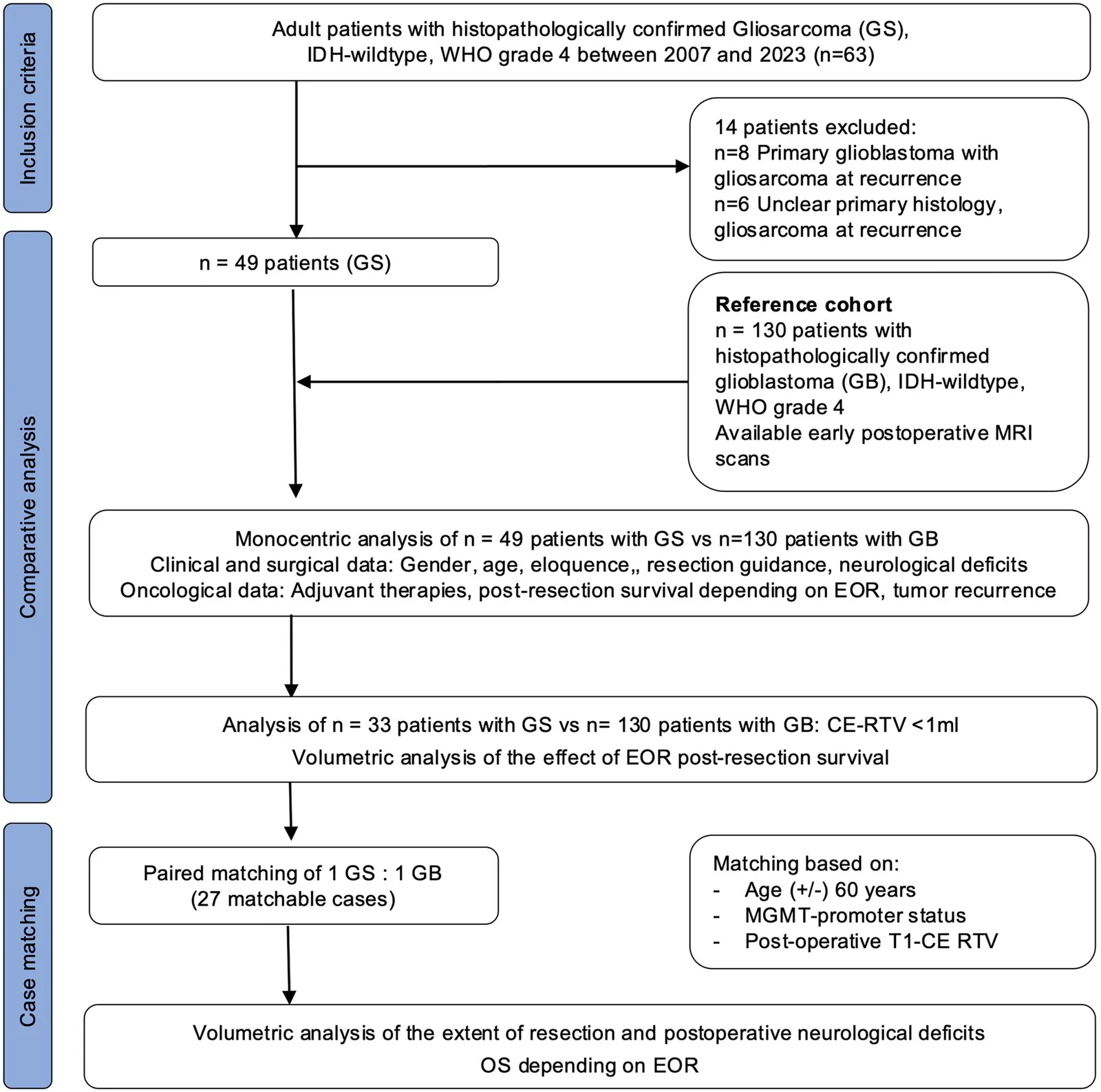
Study flow diagram and cohort selection. Flowchart summarizing identification and selection of adult patients with histopathologically confirmed gliosarcoma (GS) and glioblastoma (GB). CE-RTV, contrast-enhancing residual tumor volume; EOR, extent of resection; IDH, isocitrate dehydrogenase; MGMT, O-6-methylguanine-DNA methyltransferase.

**Table 1.** Baseline characteristics of the GS patient cohort

Variable	Value (%)
Age at first diagnosis, years (SD)	61.5 (12.2)
Mean KPS (SD)	
Preoperative	83.3 (10.2)
POD 7	81.8 (10.93)
Female gender	23 (47)
MGMT promoter methylation status (*n* = 43)	17 (34)
Localization (*n* = 49)	
Frontal	10 (20)
Temporal	22 (44)
Parietal	6 (12)
Occipital	1 (2)
Multifocal	10 (20)
Resection guidance (*n* = 45)	
5 ALA	10 (20)
iMRI	22 (44)
Neither/no resection	12 (24)
Both	1 (4)
Awake craniotomy	2 (4)
Therapy after resection^a^	
Temozolomide concomitant	28 (56)
Temozolomide adjuvant	21 (42)
Temozolomide monotherapy	1 (2)
Postoperative radiotherapy	41 (82)

Percentage values are displayed in parenthesis unless specified otherwise.

Abbreviations: 5-ALA, 5-aminolevulinic acid; iMRI, intraoperative magnetic resonance imaging; KPS, Karnofsky Performance Status; MGMT, O6-Methylguanine-DNA Methyltransferase; POD 7, postoperative day 7.

a Further adjuvant therapies have been omitted for brevity.

### Differential Effect of Surgical Radicality on Outcomes of GS and GB Patients

As a reference cohort, we included a consecutive series of 130 patients with histologically confirmed GB, WHO grade 4, treated at our institution between 2015 and 2023. Patients were consecutively enrolled and were eligible if they underwent microsurgical resection with the intention of achieving GTR and had complete pre- and postoperative volumetric MRI data available. Patients who underwent stereotactic biopsy only were excluded. To ensure comparability with the GS cohort and to specifically investigate the prognostic relevance of minimal residual disease, the present analysis was restricted to patients with a postoperative RTV <1 mL. Baseline demographic and clinical characteristics were similar between the GS and GB cohorts, especially regarding age at diagnosis (Figure 2A) and KPS, both preoperatively and at POD 7 (Figure 2B and Table 2). The use of resection-guidance adjuncts differed between cohorts: We observed a significant overrepresentation of iMRI-guidance in the GB (80%) compared to the GS (58%) cohort, and consequently, 5-ALA fluorescence was used more frequently in GS patients (24% vs 8% in GB, Figure 2C, *P* = .0175, Chi-square test). Only a minority of patients in either group received both modalities (3% GS, 2% GB). Preoperative T1-CE tumor volumes were significantly larger in GS (32.1 mL) compared with GB patients (22.37 mL, *P* = 0.021, unpaired *t*-test; Figure 3A). There was no significant difference in mean postoperative RTV between GS (0.09 mL) and GB (0.12 mL) patients on epMRI scans (*P* = .435 unpaired *t*-test; Figure 3A; Table 2). A smaller but non-significant difference in post-operative FLAIR-hyperintense volume was also evident (GS 11.55 mL vs 7.10 mL in GB, *P* = 0.063, Welch’s test). To infer on a possible modulation of GS or GB on the effect of the EOR on patient survival, we identified 33 GS patients and 130 GB patients with <1 RTV and stratified GS and GB patients by RTV (0 mL vs >0-1 mL). Here, we observed neither an overrepresentation of MGMT promoter methylation (GS, *P* > 0.999; GB, *P* = 0.453, Chi-square test; Figure 3B) nor differences in mean age or KPS at diagnosis or postoperatively in neither histological (GS vs GB) nor RTV groups (0 mL vs >0-1 mL; Figures 3C and D) were observed, although the proportion of patients treated with chemoradiation was non-significantly higher than in the complete cohort (72% vs 56%, *P* = 0.220, Chi-square test). The difference in median OS between GS (720 days) and GB (648 days) patients was not significant (Figures 4A and B). When stratified by EOR and postoperative RTV, survival patterns diverged between tumor types: In GS, patients with RTV of 0 mL demonstrated significantly improved survival compared with those with RTV of 0-1 mL (median OS 720 vs 402 days; Log-rank [Mantel–Cox] test, *P* = .022; Figure 4C). In GB, differences in RTV yielded a significant, but less pronounced, survival benefit, with median OS of 0 mL RTV being 713 vs 510 days in the RTV >0-1 mL group (*P* = .042; Figure 4D). This suggests that GS patients might derive a greater survival benefit from more radical tumor resection.

**Figure 2.**
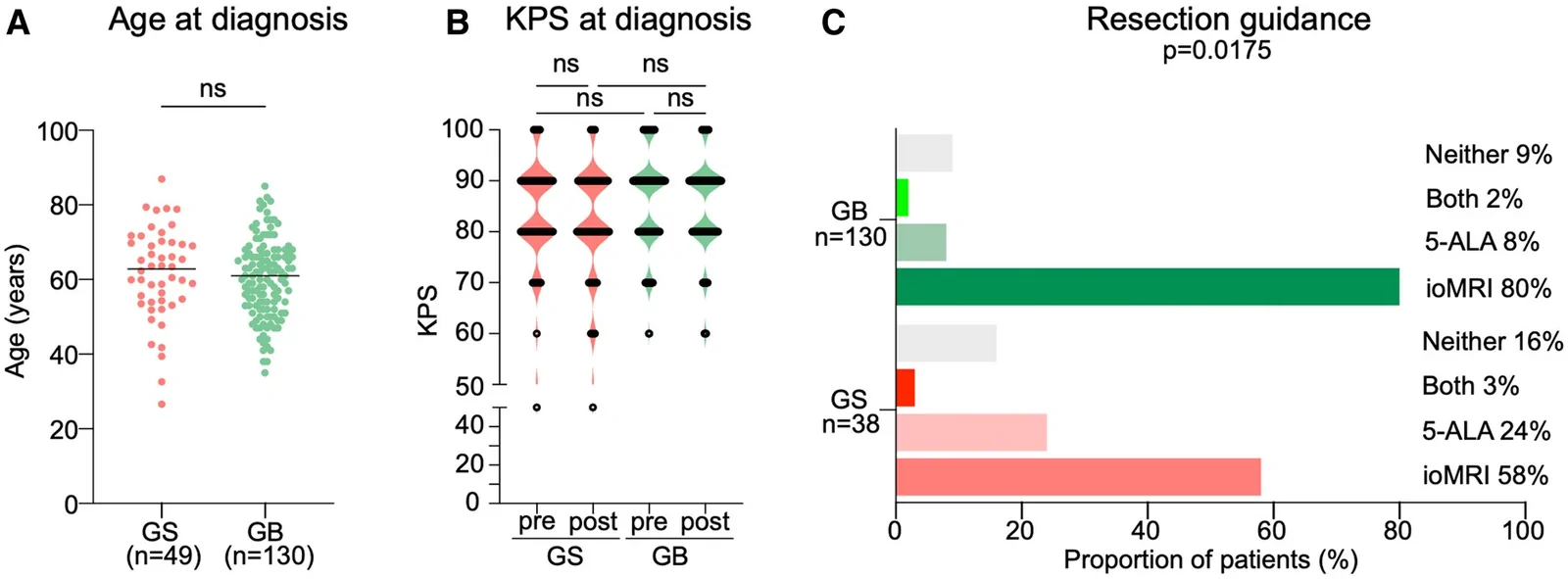
Baseline characteristics and resection guidance in GS and GB cohorts. (A): Scatter plots showing age at diagnosis for gliosarcoma (GS, *n* = 49) and glioblastoma (GB, *n* = 13). (B) Violin plots illustrating pre- and postoperative Karnofsky Performance Status (KPS) scores in GS and GB. (C) Distribution of resection-guidance modalities used in GS (*n* = 38) and GB (*n* = 130) cases (*P* = .0175, Chi-square test). Percentages indicate the proportion of patients receiving no adjunct, intraoperative MRI (iMRI), 5-aminolevulinic acid (5-ALA), or both.

**Figure 3.**
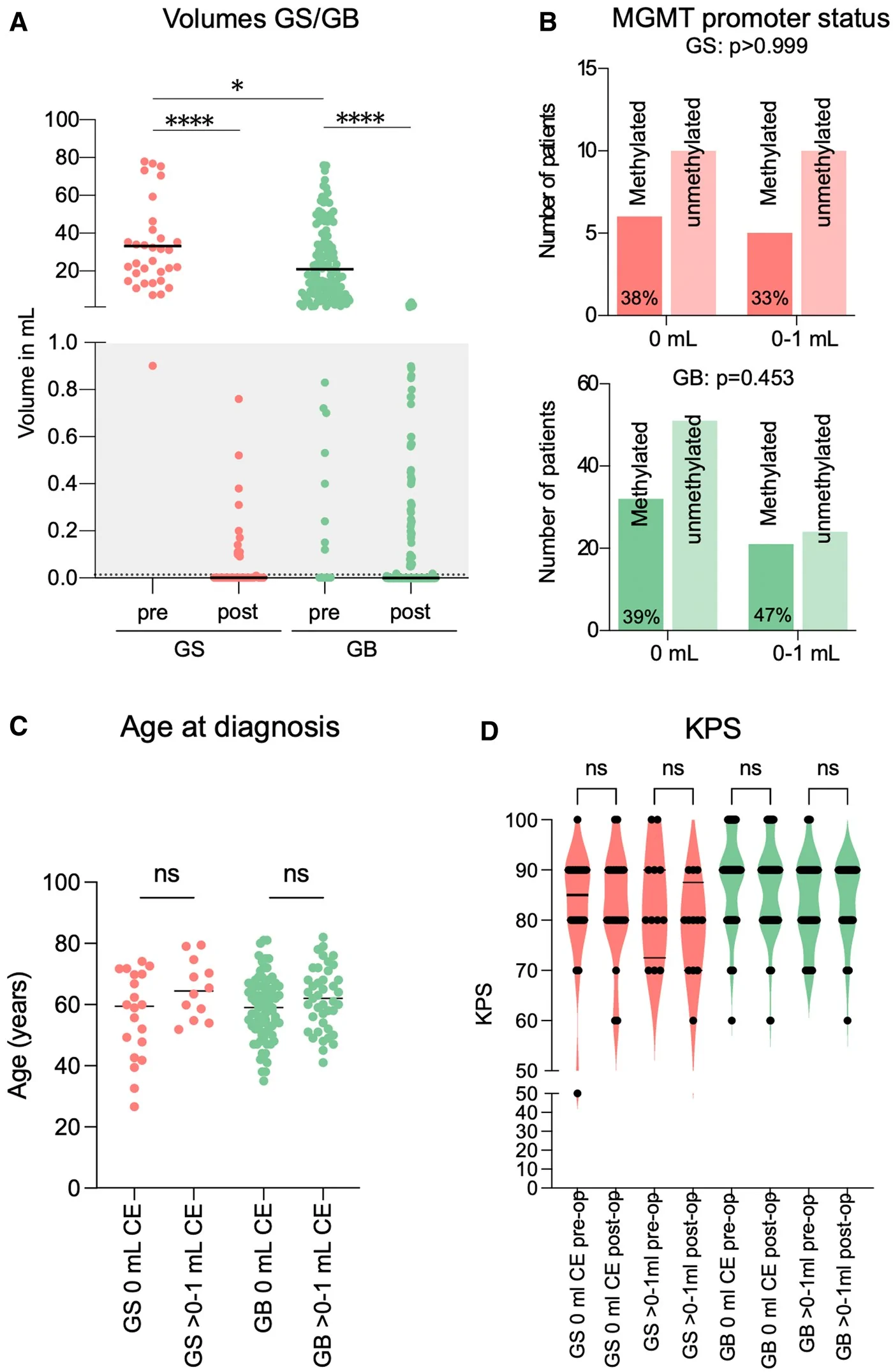
Comparison of prognostic variables between gliosarcoma (GS) and glioblastoma (GB) patients. (A) Pre- and postoperative contrast-enhancing (CE) tumor volumes in GS and GB. The lower panel highlights postoperative RTV values in the 0-1 mL range. (B) Distribution of O-6-methylguanine-DNA methyltransferase (MGMT) promoter methylation within RTV subgroups (0 mL vs 0-1 mL, *P* > .999) in GS (top) and GB (bottom, *P* = .453, Chi-square test). Percentages refer to the proportion of tumors that are methylated within each subgroup. (C) Age at diagnosis stratified by postoperative residual CE tumor volume (RTV: 0 mL vs >0-1 mL) in GS and GB patients (ns, non-significant, Welch’s *t*-test). (D) Violin plots of the Karnofsky Performance Scale (KPS) preoperatively and postoperatively in GS and GB patients, stratified by RTV (0 mL vs >0-1 mL, Welch’s *t*-test).

**Figure 4.**
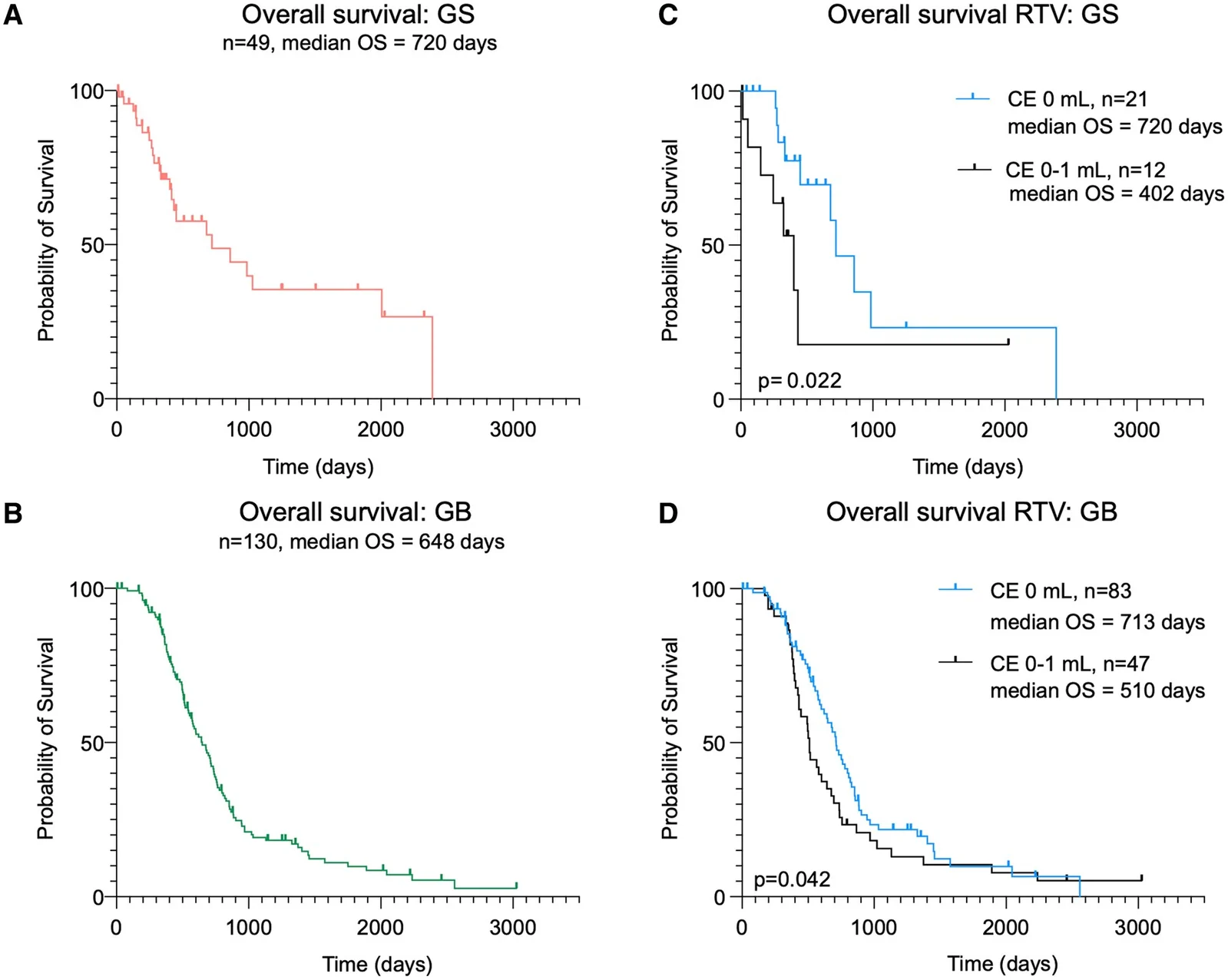
Overall survival stratified by tumor type and residual tumor volume (RTV) in gliosarcoma (GS) and glioblastoma (GB) patients. (A and B): Kaplan–Meier curves depicting overall survival in GS (*n* = 49; median overall survival [OS] 720 days) and GB (*n* = 130; median OS 648 days). (C) Survival in GS patients with RTV = 0 mL (median OS 720 days) and those with RTV 0-1 mL (median OS 402 days; *P* = .022, Logrank [Mantel–Cox] test). (D) Survival in GB patients with RTV = 0 mL vs RTV 0-1 mL (median OS 713 vs 510 days, respectively; *P* = .042, Logrank [Mantel–Cox] test).

**Table 2.** Comparative analysis of baseline characteristics in GS and GB patient cohorts with <1 mL residual tumor volume (RTV)

Variable	GS (*n* = 33)	GB (*n* = 130)	*P*-value
Gender			.844^a^
Male	20 (61)	74 (57)	
Female	13 (39)	56 (43)	
Age			.816^b^
Mean (SD)	59.8 (13.1)	60.3 (10.4)	
Resection guidance			.029^a^
iMRI	18 (54)	101 (78)	
5-ALA	8 (24)	10 (8)	
Both	1 (3)	3 (2)	
Neither	6 (18)	16 (12)	
Tumor volumetrics mean (SD) in mL			
preOP T1-CE	32.12 (21.97)	22.37 (21.34)	.021^b^
preOP T2-FLAIR	22.26 (12.64)	16.28 (18.35)	.079^b^
postOP T1-CE	0.09 (0.17)	0.12 (0.23)	.435^b^
postOP T2-FLAIR	11.55 (8.50)	7.10 (12.96)	.063^b^
Awake craniotomy	1 (3)	17 (13)	.129^c^
MGMT promoter methylation status			.697^a^
Methylated	10 (30)	53 (41)	
Non-methylated	17 (51)	76 (58)	
Unknown	6 (19)	1 (1)	
Tumor localization			.604^a^
Frontal	9 (27)	35 (27)	
Temporal	12 (36)	48 (37)	
Parietal	5 (15)	12 (9)	
Occipital	0	9 (7)	
Insular	0	2 (2)	
Deep	0	1 (1)	
Eloquent	16 (53)	33 (25)	.444^a^
Mean KPS			
Pre-operative	83.1 (10.6)	86.5 (9)	.071^b^
POD 7	81.3 (10.4)	85.1 (8.6)	.032^b^
3 months after surgery	75.8 (26)	79.1 (26.2)	.550^b^
Adjuvant therapy after resection^d^			.870^a^
Temozolomide concomitant	24 (72)	111 (85)	
Temozolomide adjuvant	19 (58)	93 (70)	
Temozolomide monotherapy	2 (6)	7 (5)	
Postoperative Radiotherapy	27 (84)	118 (91)	.088^a^
Re-resection at recurrence	17 (52)	56 (43)	.380^a^

Percentage values are displayed in parenthesis unless specified otherwise.

Abbreviations: 5-ALA, 5-aminolevulinic acid; GB, glioblastoma; GS, gliosarcoma; iMRI, intraoperative magnetic resonance imaging; KPS, Karnofsky Performance Status; MGMT, O6-methylguanine-DNA methyltransferase; POD 7, postoperative day 7; postOP, postoperative; preOP, preoperative; T1-CE, T1-weighted contrast-enhancing; T2-FLAIR, T2-weighted fluid-attenuated inversion recovery.

a Chi-square test.

b two-tailed *t*-test.

c Fisher’s exact test.

d Further adjuvant therapies have been omitted for brevity.

### A Matched Cohort of GS and GB Patients Reveals Specific Effects of Surgical Radicality and Resulting Survival

To validate the differences in patient survival between GS and GB patients, we matched 27 GS patients with RTV <1 mL to the GB cohort in a 1:1 fashion (Table 3). A total of 54 patients were included in this matched cohort. Baseline demographic characteristics were well balanced, with no significant differences in gender distribution (male: 59% vs 56%, *P* = .659, Chi-square test) or age at surgery (60.8 ± 12.8 vs 62.0 ± 10.3 years, *P* = .706, Student’s *t*-test). Surgical strategies still differed between groups, with iMRI used more frequently in the GB cohort (85% vs 52%) and 5-ALA more commonly employed in GS patients (30% vs 7%, *P* = .049, Chi-square test). Resection via awake craniotomy was uncommon and comparable between groups (4% vs 11%, *P* = .317, Chi-square test). Preoperative CE tumor volumes were similar between GS and GB patients (33.0 ± 22.0 mL vs 24.9 ± 26.9 mL, *P* = .227). In contrast, preoperative T2-FLAIR volumes were significantly larger in the GS cohort (22.8 ± 13.1 mL vs 15.8 ± 11.0 mL, *P* = 0.038, Student’s *t*-test). Postoperatively, RTV was minimal and comparable between groups (0.01 ± 0.18 mL vs 0.13 ± 0.24 mL, *P* = 0.568, Student’s *t*-test). In comparison, residual T2-FLAIR tumor volume was non-significantly higher in GS patients (11.6 ± 8.8 mL vs 7.6 ± 8.5 mL, *P* = 0.096, Student’s *t*-test). O-6-methylguanine-DNA methyltransferase promoter methylation status was identical in both cohorts (37% methylated, *P* = 1.0), and tumor localization and eloquent brain involvement did not differ significantly. Preoperative Karnofsky Performance Score (KPS) was comparable between groups (82.7 ± 11.2 vs 84.4 ± 8.5, *P* = 0.522, Student’s *t*-test; Table 3); however, at hospital discharge, GS patients exhibited a significantly lower mean KPS than GB patients (80.0 ± 10.0 vs 85.6 ± 7.5, *P* = .035, Student’s *t*-test; Table 3), with no significant difference observed at postoperative day 7 (*P* = .142, Student’s *t*-test). Rates of adjuvant temozolomide, concomitant chemotherapy, and radiotherapy were comparable between groups (all *P* > 0.15, Chi-square test; Table 3). Survival analysis demonstrated a numerically longer median OS in GS patients compared with GB patients, although this difference did not reach statistical significance (1028 vs 621 days; Log-rank (Mantel–Cox) test; *P* = .08; Figure 5A). Stratification by residual contrast-enhancing tumor volume (CE-RTV) showed longer median OS for patients with complete resection (CE-RTV = 0) compared with those with minimal residual enhancement (CE-RTV 0-1 mL) in both GS (1170 vs 572 days; *P* = 0.516, Log-rank [Mantel–Cox] test; Figure 5B) and GB (680 vs 510 days Log-rank [Mantel–Cox] test; *P* = 0.463; Figure 5C). Again, here, the difference in survival stratified by EOR was more pronounced in GS patients, with a similar rate of re-resection surgery at recurrence 52% for GS and 43% for GB (*P* = 0.380, Chi-square; Table 2). Altogether, these findings indicate that, despite comparable baseline and adjuvant treatment characteristics, GS patients exhibited a greater benefit from a more radical resection of CE tumor within this matched cohort.

**Figure 5.**
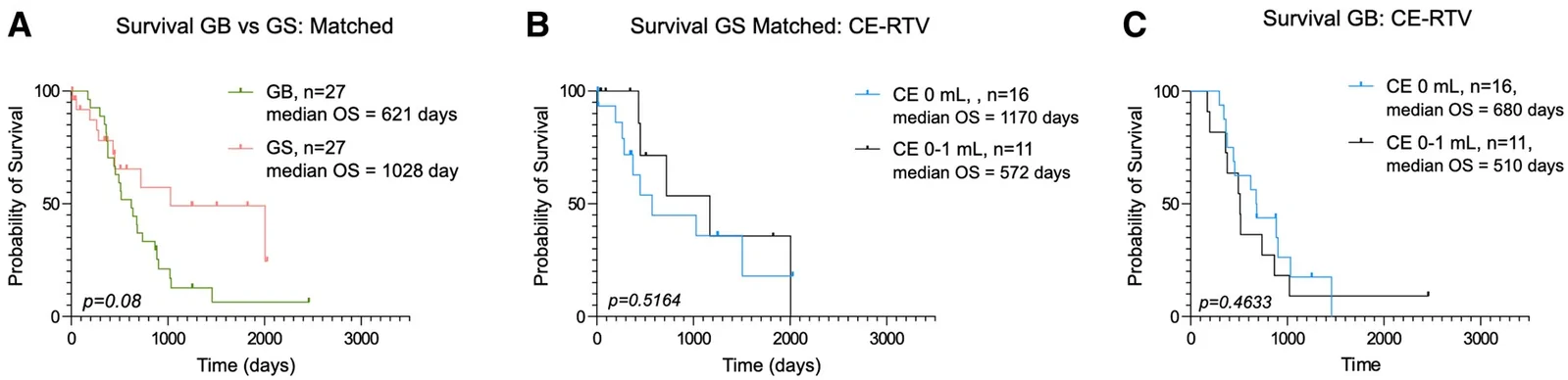
Overall survival analyses in a matched gliosarcoma (GS) and glioblastoma (GB) cohort. (A) Kaplan–Meier analysis comparing overall survival between matched GS (*n* = 27) and GB (*n* = 27) patients (1028 vs 621 days, *P* = .08, Logrank [Mantel–Cox] test). (B) Overall survival of GS patients stratified by residual contrast-enhancing tumor volume (CE-RTV), comparing complete resection (CE-RTV = 0, *n* = 16) with minimal residual enhancement (CE-RTV 0-1 mL, *n* = 11); median overall survival (OS) 1,170 vs 572 days (*P* = .516, Logrank [Mantel–Cox] test). (C) Overall survival of GB patients stratified by CE-RTV. CE-RTV = 0 (*n* = 16) vs CE-RTV 0-1 mL (*n* = 11); 680 vs 510 days; *P* = .463, Logrank (Mantel–Cox) test.

**Table 3.** Comparative analysis of baseline characteristics in matched GS and GB patient cohorts with <1 mL residual tumor volume (RTV)

Variable	GS (*n* = 27)	GB (*n* = 27)	*P*-value
Gender			
Male	16 (59)	15 (56)	>.999^a^
Female	11 (37)	12 (44)	
Age			
Mean (SD)	60.8 (12.8)	62 (10.3)	.706^b^
Resection guidance			.073^a^
iMRI	14 (52)	23 (85)	
5-ALA	8 (30)	2 (7)	
Both	1 (4)	0 (0)	
Neither	3 (11)	2 (7)	
Unknown	1 (4)	0	
Tumor volumetrics mean (SD) in mL			
preOP T1-CE	33.0 (22.0)	24.9 (26.9)	.227^b^
preOP T2-FLAIR	22.8 (13.1)	15.8 (11.0)	.038^b^
postOP T1-CE	0.01 (0.18)	0.13 (0.24)	.568^b^
postOP T2-FLAIR	11.6 (8.82)	7.60 (8.50)	.096^b^
Awake craniotomy	1 (4)	3 (11)	.317^a^
MGMT promoter methylation status			1^a^
Methylated	10 (37)	10 (37)	
Non-methylated	17 (63)	17 (63)	
Tumor localization			
Frontal	7 (26)	11 (41)	.205^a^
Temporal	10 (37)	7 (26)	
Parietal	3 (11)	2 (7)	
Occipital	0 (0)	3 (11)	
Multifocal	7 (26)	3 (11)	
Eloquent	12 (44)	17 (63)	1^a^
Mean KPS before surgery	82.7 (11.2)	84.44 (8.5)	.522^b^
Mean KPS post-operatively			
POD 7	80 (10)	85.6 (7.5)	.035^b^
3 months	72.5 (28.8)	83 (12.7)	.142^b^
Adjuvant therapy after resection^c^			
Temozolomide concomitant	19 (70)	19 (70)	1^a^
Temozolomide adjuvant	19 (70)	23 (85)	.159^a^
Temozolomide monotherapy	1 (4)	1 (4)	1^a^
Postoperative radiotherapy	21 (78)	25 (93)	.204^a^

Patient percentages are displayed in parenthesis unless specified otherwise. Number of patients (%), Mean (SD).

Abbreviations: 5-ALA, 5-aminolevulinic acid; GB, glioblastoma; GS, gliosarcoma; iMRI, intraoperative magnetic resonance imaging; KPS, Karnofsky Performance Status; MGMT, O6-methylguanine-DNA methyltransferase; POD 7, postoperative day 7; postOP, postoperative; preOP, preoperative; T1-CE, T1-weighted contrast-enhancing; T2-FLAIR, T2-weighted fluid-attenuated inversion recovery.

a Chi-square test.

b Two-tailed *t*-test.

c Further adjuvant therapies have been omitted for brevity

## Discussion

In this study of 49 GS patients, we demonstrated that despite larger preoperative CE tumor volumes, GTR rates were comparable to those achieved in a reference cohort of 130 GB patients. Importantly, complete resection conferred a more substantial survival benefit in GS patients than in GB patients. Gliosarcoma patients achieving complete resection (0 mL RTV) had a median OS of 720 days compared to 402 days for those with minimal residual disease (>0 to <1 mL)—a difference of 318 days. In contrast, the corresponding survival difference in GB patients was less pronounced at 203 days (713 vs 510 days). These findings were validated through matched cohort analysis that controlled for baseline demographics and MGMT promoter methylation status. Indeed, both histological subtypes demonstrated improved outcomes with a greater EOR.

In GB, distinct methylation subclasses have been shown to influence the survival benefit associated with greater EOR.^7^^,^^19^^,^^20^ Analogously, given potential intrinsic biological differences between GB and GS based on histology, it is conceivable that GS, as a tumor, may similarly modulate the impact of residual CE tumor volume on survival. Indeed, differences between both “entities” regarding clinical outcomes, resistance to therapy, or immune invasion have been observed in the past.^16^^,^^21^^,^^22^ In a wider context and in line with the literature, these findings reinforce the critical importance of maximizing the EOR when feasible in any given GB histological subtype.^23^^,^^24^

Interestingly, we observed an overrepresentation of 5-ALA use for resection guidance in patients with GS compared to those with GB. Our center’s previous heavy reliance on iMRI for resection guidance in cases of suspected gliomas, until evidence of non-inferiority of both modalities had been established,^25^^,^^26^ could provide a potential explanation for this observation. Supporting this, the circumscribed and well-demarcated appearance on preoperative imaging could have led to the suspicion of metastases rather than primary glial tumors in GS patients and hence for surgery planning without iMRI guidance, with fluorescence-guidance (5-ALA) serving as a “backup” modality. There have been distinctions in the past between both entities in terms of radiological appearance and surgical management, with GS being described as more compact and GB as less circumscript,^16^^,^^27-29^ but equally conflicting evidence in the literature.^30^^,^^31^

As a high-volume neuro-oncology referral center, we were able to include 49 GS patients, representing a relatively large cohort, which is a major strength of this study, giving this rare tumor subtype accounting for only ∼2% of all GB cases.^14^^,^^15^ Although GS and GB cannot currently be distinguished reliably pre- or intraoperatively, and maximal safe resection remains the mainstay of care for both entities, emerging intraoperative image- and molecular-based diagnostic technologies may eventually enable real-time differentiation between GS and GB.^32-34^ In cases where further resection is limited by anticipated neurological morbidity due to eloquent (residual) tumor location, intraoperative knowledge of tumor subtype could provide an additional biological context for weighing the potential oncological benefit of extending resection against the risk of permanent functional impairment.^35^^,^^36^

Several limitations must be acknowledged. First, our study did not investigate the utility of supramarginal resection of FLAIR-positive regions compared to standard GTR, as this has been explored in larger multicenter GB cohorts.^4^^,^^23^ In addition, owing to a small sub-cohort sample size, this study also did not distinguish between primary and secondary GS (as a recurrence), which have been reported to differ in outcome, with secondary GS showing a more aggressive nature.^37^^,^^38^ In addition, the apparent differences in survival benefit associated with a more extensive resection in GB and GS patients cannot be fully disentangled from differences in postoperative treatment allocation, as patients achieving lower residual tumor burden or RANO 1/2 resection were also more likely to proceed to adjuvant chemoradiation. Differences in survival between GS and GB patients in the matched cohort analysis were not significant and may be confounded by variables not accounted for by case matching. In addition, for differences were observed in the matched cohort, they did not reach statistical significance, suggesting a potential risk of type II errors. However, these observed differences are still useful for future hypothesis synthesis. A further limitation of this study is that analyses were restricted to patients with postoperative RTV <1 mL to specifically investigate the biological impact of minimal residual CE tumor rather than to compare the overall GS and GB populations. Although this reduced treatment-related heterogeneity, it limits the generalizability of our findings beyond patients with minimal residual disease. Finally, while our findings suggest a stronger dependency on EOR in GS, the underlying biological explanation remains unclear. No molecular or histopathological hallmark currently differentiates GS in a way that directly explains this disproportionate sensitivity to residual CE tumor, and future studies incorporating molecular subclassification and potentially more elaborate molecular genetic characterization are needed.

Future research should focus on validating these findings in multicenter cohorts, exploring the role of FLAIR-based resection strategies, and incorporating more comprehensive assessments of postoperative functional outcomes. Such approaches may help refine surgical decision-making and improve individualized treatment strategies.

## Conclusion

This study demonstrates that, despite larger preoperative tumor volumes in this retrospective cohort, comparable rates of complete resection can be achieved in patients with GS and GB. Furthermore, our findings suggest that the prognostic significance of complete, compared with near-total, resection may differ between these histological subtypes. If confirmed in future studies, advances in intraoperative diagnostic technologies enabling real-time differentiation between GS and GB may facilitate histology-informed surgical decision-making in selected cases where the EOR is limited by functional considerations.

## Data Availability

The data are available upon reasonable request to the corresponding author.
